# Autonomic responses during bladder hydrodistention under general versus spinal anaesthesia in patients with interstitial cystitis/bladder pain syndrome: a randomized clinical trial

**DOI:** 10.1038/s41598-023-36537-y

**Published:** 2023-06-07

**Authors:** Yoon Jung Kim, Hyun-Kyu Yoon, Yu Jin Kang, Seung-June Oh, Min Hur, Hee-Pyoung Park, Hyung-Chul Lee

**Affiliations:** 1 Department of Anesthesiology and Pain Medicine, Seoul National University College of Medicine, Seoul National University Hospital, 101 Daehak-ro, Jongno-gu, Seoul, 03080 Republic of Korea; 2 Department of Urology, Pohang St Mary’s Hospital, Pohang-si, Gyeongsangbuk-do South Korea; 3 Department of Urology, Seoul National University College of Medicine, Seoul National University Hospital, Seoul, South Korea; 4 Department of Anesthesiology and Pain Medicine, Ajou University College of Medicine, Suwon, South Korea

**Keywords:** Bladder, Medical research, Urology

## Abstract

Blocking the abrupt increase in systolic blood pressure associated with autonomic response during bladder hydrodistention in patients with interstitial cystitis/bladder pain syndrome (IC/BPS) is essential for patient safety. We conducted this study to compare autonomic responses during bladder hydrodistention in patients with IC/BPS under general and spinal anaesthesia. Thirty-six patients were randomly allocated to a general anaesthesia (GA, n = 18) or a spinal anaesthesia (SA, n = 18) group. Blood pressure and heart rate were measured continuously and ΔSBP, defined as maximum increases in SBP during bladder hydrodistention from baseline, was compared between groups. Heart rate variability was analysed using electrocardiograms. The post-anaesthesia care unit assessed postoperative pain using a numeric (0–10) rating scale. Our analyses yield a significantly greater ΔSBP (73.0 [26.0–86.1] *vs.* 2.0 [− 4.0 to 6.0] mmHg), a significantly lower root-mean-square of successive differences in heart rate variability after bladder hydrodistention (10.8 [7.7–19.8] vs. 20.6 [15.1–44.7] ms), and significantly higher postoperative pain scores (3.5 [0.0–5.5] *vs.* 0.0 [0.0–0.0]) in the GA compared to the SA group. These findings suggest that SA has advantages over GA for bladder hydrodistention in preventing an abrupt increase in SBP and postoperative pain in IC/BPS patients.

## Introduction

Interstitial cystitis/bladder pain syndrome (IC/BPS) is a chronic pain syndrome that causes bladder pain associated with bladder filling and is commonly accompanied by urinary symptoms in the absence of infection and other aetiology^1^. According to a community survey study, 2.7–6.5% of women in the United States have symptoms consistent with IC/BPS; however, the condition is often underdiagnosed and undertreated^2^. While the pathophysiology of IC/BPS has not been fully elucidated, a deficiency of glycosaminoglycan covering the urothelium surface, immunological reactions, activated mast cells, neural changes, and inflammation have been suggested^3^.

Bladder hydrodistention is not only a diagnostic tool, but also a treatment option for patients with IC/BPS. Although it is non-specific, diagnostic information on, for example, Hunner’s lesions or mucosal rainy bleeding can be obtained through cystoscopy with bladder hydrodistention^4^. Additionally, bladder hydrodistention can improve the symptoms in patients refractory to conservative treatments such as medication and behavioural therapy^5^. Approximately half of the patients with IC/BPS who undergo bladder hydrodistention show improvements in long-term outcomes^6, 7^. However, since marked autonomic responses, such as increased blood pressure, are observed during bladder hydrodistention in IC/BPS patients^8^. adequate anaesthesia that can block the autonomic response is essential for patient safety. According to the Japanese guideline, spinal anaesthesia is a recommended anaesthetic method for bladder hydrodistention; however, the supporting evidence is contradictory^9^.

In a retrospective study with a limited number of patients, autonomic responses during bladder hydrodistention were greater in patients under general anaesthesia than in those under spinal anaesthesia^10^. Spinal anaesthesia can block all sensory, motor, and autonomic nerve transmission and^11^ lower serum concentration of catecholamines^12^. In contrast, some sensory responses and autonomic reflexes are preserved during general anaesthesia, even with the loss of consciousness^13^. Therefore, general anaesthesia may not be sufficient to block autonomic responses to bladder hydrodistention in patients with IC/BPS^14–16^. However, no prospective study has compared anaesthetic techniques in terms of autonomic responses during bladder hydrodistention in patients with IC/BPS.

In this study, we aimed to compare changes in systolic blood pressure (SBP) during bladder hydrodistention in patients with IC/BPS under general and spinal anaesthesia. We hypothesized that the change would be less prominent in patients under spinal anaesthesia.

## Materials and methods

### Ethical approval

This study was approved by the Institutional Review Board of Seoul National University Hospital (approval number: 1806-039-949, date: July 10, 2018) and registered in the Korean national registry of clinical trials before patient recruitment (number: KCT0003225, registration date: 28/09/2018), All patients provided written informed consent. We conducted this study in accordance with the Helsinki Declaration and the Good Clinical Practice guidelines and reported the findings based on the applicable Consolidated Standard of Reporting Trials guidelines. A reporting guideline checklist is included in the Supplementary Information file.

### Population

All adult patients who were diagnosed with IC/BPS and scheduled to undergo bladder hydrodistention for either diagnostic or therapeutic purposes at Seoul National University Hospital since October 2018 to December 2021 were eligible for this study. Among these patients, those aged 20 or older who had an American Society of Anesthesiologists (ASA) physical status of I–III were included. The exclusion criteria were contraindications for general anaesthesia or spinal anaesthesia (e.g., severe cardiopulmonary dysfunction, coagulopathy, taking anticoagulants, septicaemia, skin infection near the lumbar puncture site, spinal cord lesion, increased intracranial pressure, neurologic disorders, or spinal deformities).

### Randomization

Before patient recruitment, an anaesthesiologist who was not involved in this study prepared a random allocation sequence with a one-to-one ratio using a computer-generated randomized table. Patients were allocated to two groups: the general anaesthesia (GA) group and the spinal anaesthesia (SA) group. The clinical research coordinator who was blinded to this study managed the randomization table and notified the attending anaesthesiologist of the group allocation on the day of surgery.

### Protocol

After entering the operation room, patients underwent standard monitoring procedures, including assessments for percutaneous oxygen saturation and non-invasive blood pressure, as well as electrocardiography.

In the GA group, a bolus injection of 1.5–2.0 mg/kg propofol and the target-controlled infusion of remifentanil with an effect-site concentration of 4.0 ng/mL were used for anaesthesia induction. After confirming the loss of consciousness, 0.6 mg/kg rocuronium was administered to facilitate insertion of a supraglottic airway device. After appropriate placement of the supraglottic airway device, a 20-gauge catheter was inserted into the radial artery to monitor invasive blood pressure continuously. Anaesthesia was maintained using sevoflurane and remifentanil. The depth of anaesthesia was monitored using the bispectral index (Medtronic, Ireland), with a target of 40–60 during the procedure.

The effect-site concentration of remifentanil was increased up to 4–6 ng/mL to block autonomic nervous system responses 90 s before initiating bladder hydrodistention. If the mean blood pressure (MBP) dropped below 65 mmHg, the target effect-site concentration of remifentanil was lowered by 1 ng/mL.

In the SA group, spinal anaesthesia was performed at the lateral decubitus position, using a 25-gauge Quincke needle under aseptic drapes. Considering the height of the patient, 12–14 mg of 0.5% bupivacaine was injected intrathecally. The accepted sensory block level was T10 or higher. Subsequently, radial artery catheterization was performed to monitor invasive blood pressure.

In both groups, a rescue drug (ephedrine 5–10 mg, phenylephrine 20–50 µg) was administered if the MBP dropped below 65 mmHg, based on the decision of the attending anaesthesiologist. If the patient’s heart rate (HR) fell below 45 beats/min, 0.5 mg of atropine was injected intravenously. Midazolam or dexmedetomidine was administered intravenously if patients in the SA group required sedation.

### Operational procedure

After the induction of anaesthesia, patients were placed in the lithotomy position. A 30°-angled cystoscope was inserted through the urethra to drain the bladder of all urine. Hunner’s lesions were identified and thoroughly inspected under the cystoscope, and their location, number, and area were noted. Bladder hydrodistention was then performed by filling the bladder with normal saline by gravity from a height of 80 cm above the pubic symphysis. Maximum filling in the bladder was maintained for 8 min, and the maximum bladder volume was recorded. Changes in Hunner’s lesions and glomerulation were observed while the saline was drained. Bladder biopsy was performed and the bleeding area, including the mucosal crack, was cauterized to control bleeding. The hyperaemic and congestion areas with Hunner’s lesions were also coagulated. After confirming that there was no bleeding, the surgeon inserted a 6.6-mm three-way catheter for postoperative continuous irrigation. A bimanual examination was performed to check whether any tumour was present in the pelvic cavity, and the surgery was terminated. The entire procedure was performed by a single surgeon (SJO) with the assistance of an attending urologist (YJK).

### Measurements

The vital signs and electrocardiogram were recorded during the patient’s stay in the operating room using the Vital Recorder (version 1.8.19.5; https://vitaldb.net/vital-recorder, accessed Feb 21, 2018) with a resolution of 0.5 and 500 samples per second, respectively^17^.

The primary outcome measure was ΔSBP, defined as the maximum increase in SBP during bladder hydrodistention from baseline (measured 5 min after the induction of anaesthesia). The secondary outcome measures were the maximum increase in MBP, diastolic blood pressure (DBP), and HR during bladder hydrodistention from baseline. Standard heart rate variability (HRV) measures^18^ such as root-mean-square of successive differences (RMSSD), the standard deviation of the normal-to-normal interval (SDNN), and the high-frequency/low-frequency ratio, were calculated using the Vital Recorder on 5-min electrocardiograms recorded before and after bladder hydrodistention, respectively.

The recorded cystoscopic descriptions included Hunner’s lesions, bleeding patterns, and the maximal bladder volume. Postoperative pain scores were evaluated in the post-anaesthesia care unit (PACU) based on a numeric rating scale (NRS), with 0 reflecting “no pain” and 10 “the worst pain imaginable.” If the patient complained of pain in the PACU, 25 µg of fentanyl was administered intravenously. The number of patients who required analgesics in the PACU was also recorded.

### Statistical analysis

The data are presented as numbers (proportions) for categorical variables and means (standard deviations) or medians (interquartile ranges) for continuous variables, depending on the normality of their distributions evaluated with the Shapiro–Wilk test. We used the Pearson’s chi-square test or Fisher’s exact test to compare categorical variables and Student’s *t*-tests and the Mann–Whitney *U* test to compare continuous variables with normal and skewed distributions, respectively.

Since rescue vasopressor administered before bladder hydrodistention can cause an elevation of blood pressure, we performed a post hoc subgroup analysis on the patients who were not administered rescue vasopressors.

All statistical analyses were conducted using SPSS statistical software for Windows, version 25.0 (IBM, Armonk, NY, USA) and R version 4.0.3 (The R Foundation for Statistical Computing, Vienna, Austria). We considered a P value less than 0.05 as statistically significant.

### Sample size calculation

A previous retrospective study reported a ΔSBP in patients with IC/BPS of 56.26 ± 30.38 mmHg under general anaesthesia^10^. We considered a mean difference in ΔSBP between the two anaesthesia groups of 30 mmHg or more statistically significant. We estimated a required minimum sample size of 16 patients in each group, based on α = 0.05 and power (1 − β) = 0.8. Considering a 10% dropout rate, we recruited 18 patients for each group.

## Results

**Figure 1:**
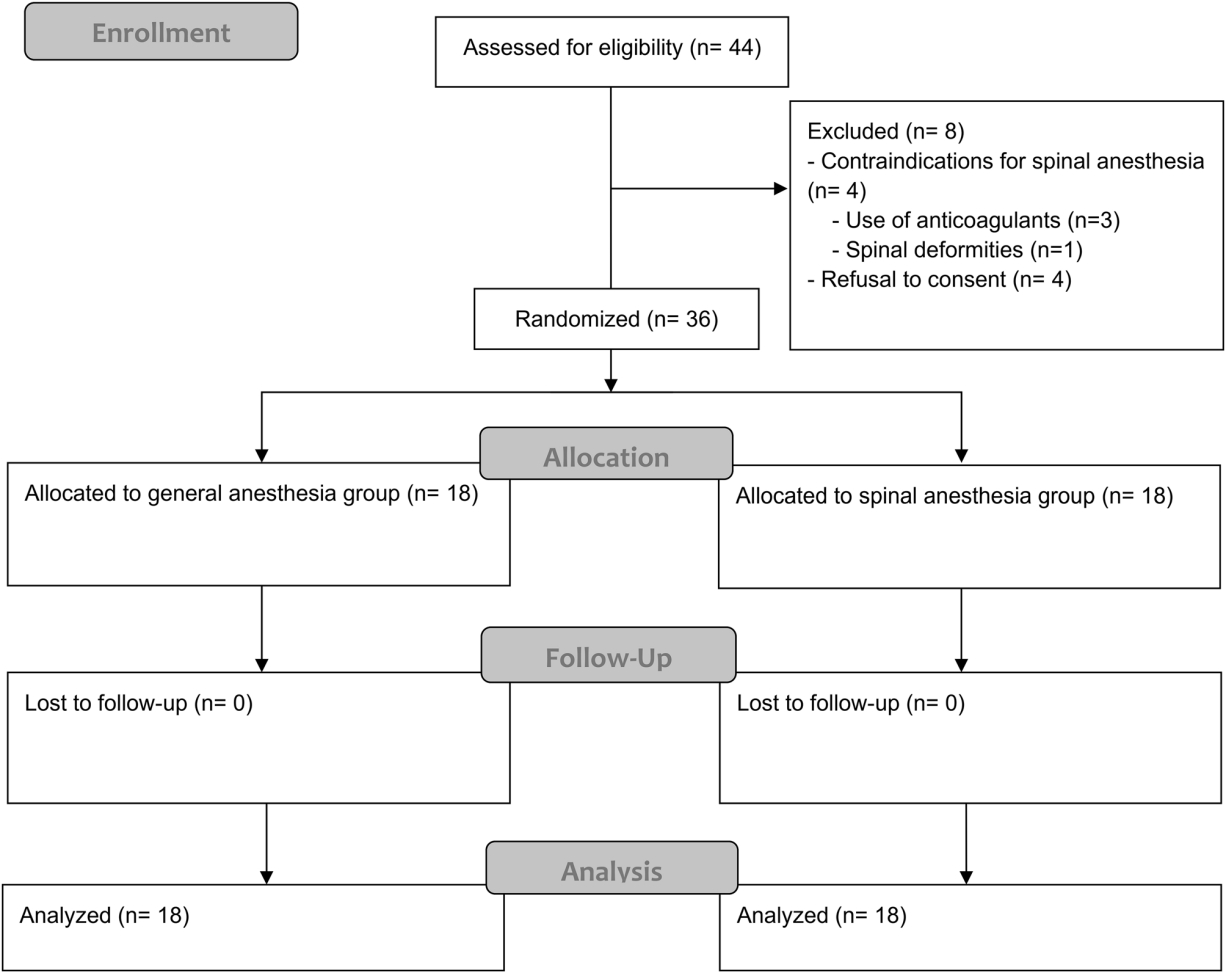
CONSORT diagram.

The data of 36 patients who underwent bladder hydrodistention between October 2018 and December 2021 at our institution were entered in our analyses (see Fig. 1).

The patients had been randomly allocated to the GA and the SA group. Table 1 lists the patients’ characteristics, 72-h voiding diaries, and intraoperative findings.

**Table 1:** General patient characteristics.

	GA group	SA group	P value
	(n = 18)	(n = 18)	
Sex (female)	16 (88.9%)	15 (83.3%)	1.000
Age (y)	64.3 ± 8.9	61.9 ± 8.8	0.432
Weight (kg)	58.6 ± 7.4	59.3 ± 7.5	0.776
Height (cm)	157.5 ± 4.7	156.6 ± 8.0	0.705
BMI (kg/m^2^)	23.7 ± 3.5	24.2 ± 2.4	0.655
ASA-PS classification			1.000
1	4 (22.2%)	3 (16.7%)	
2	14 (77.8%)	15 (83.3%)	
Preoperative features			
24-h urine amount (mL)	1686 [1447–1740]	1408 [1303–1775]	0.382
Nocturnal urine amount (mL)	552 [417–626]	490 [345–511]	0.144
Nocturnal polyuria index	30.7 [30.0–34.9]	30.9 [21.6–36.5]	0.746
Number of total voiding	14.3 [12.5–20.0]	12.3 [10.5–18.0]	0.207
Number of daytime voiding	11.0 [9.6–17.0]	9.6 [8.0–13.0]	0.248
Number of nocturnal voiding	3.3 [2.3–5.0]	2.3 [1.6–5.0]	0.551
Maximal bladder capacity (mL)	211 ± 79	184 ± 67	0.301

GA, general anaesthesia; SA, spinal anaesthesia; BMI, body mass index; ASA-PS, American Society of Anesthesiologists physical status.

Hemodynamic and autonomic responses are described in Table 2.

**Table 2:** Changes in hemodynamic responses and heart rate variability during bladder hydrodistention between the two groups.

	GA group (n = 18)	SA group (n = 18)	Median, mean, or proportion difference [95% CI]	P value
Differences between max and baseline				
ΔSBP (mmHg)	73.0 [26.0–86.0]	2.0 [− 4.0 to 6.0]	− 71.0 [− 84.0, − 30.5]	< 0.001
SBP increase > 20%	14 (77.8%)	0 (0.0%)	− 77.8% [− 91.0%, − 48.9%]	< 0.001
ΔDBP (mmHg)	42.5 [24.0–56.0]	3.0 [− 2.0 to 6.0]	− 39.5 [− 53.0, − 22.0]	< 0.001
ΔMBP (mmHg)	42.9 ± 28.3	− 1.4 ± 29.0	− 44.4 [− 63.8, − 25.0]	< 0.001
ΔHR (beat per min)	18.6 ± 19.6	3.5 ± 14.9	− 15.1 [− 26.9, − 3.2]	0.014
Baseline values^a^				
SBP (mmHg)	96.5 [90.0 − 110.0]	140.5 [131.0–172.0]	47.9 [31.1, 64.7]	< 0.001
DBP (mmHg)	53.7 ± 12.7	75.3 ± 9.8	21.6 [13.9, 29.2]	< 0.001
MBP (mmHg)	70.6 ± 13.7	100.3 ± 18.3	29.7 [18.8, 40.7]	< 0.001
HR (beat per min)	67.1 ± 11.9	70.8 ± 14.4	3.8 [− 5.2. 12.7]	0.396
Maximum values during hydrodistention				
SBP (mmHg)	159.7 ± 38.0	145.7 ± 22.7	− 14 [− 35.4, 7.4]	0.191
DBP (mmHg)	93.2 ± 26.5	74.6 ± 10.0	− 18.6 [− 32.5, − 4.8]	0.011
MBP (mmHg)	113.5 ± 30.2	104.7 ± 20.4	− 8.9 [− 26.6, 8.9]	0.320
HR (beat per min)	85.6 ± 19.5	74.3 ± 15.4	− 11.3 [− 23.2, 0.6]	0.063
HRV before bladder hydrodistention				
RMSSD (ms)	12.1 [6.5–32.7]	19.3 [13.2–29.4]	7.2 [− 22.1, 19.4]	0.233
SDNN (ms)	14.1 [8.8–26.3]	20.5 [15.2–32.8]	6.4 [− 8.4, 20.4]	0.486
LF/HF ratio	2.5 [1.3–6.7]	2.3 [0.5–12.8]	− 0.2 [− 5.8, 7.0]	0.935
HRV after bladder hydrodistention				
RMSSD (ms)	10.8 [7.7–19.8]	20.6 [15.1–44.7]	9.8 [− 2.6, 34.9]	0.045
SDNN (ms)	14.4 [10.0–22.3]	18.5 [11.8–38.3]	4.2 [− 6.2, 23.1]	0.367
LF/HF ratio	1.2 [0.3–11.0]	1.4 [0.4–3.5]	0.1 [− 7.3, 1.9]	0.787

GA, general anaesthesia; SA, spinal anaesthesia; SBP, systolic blood pressure; DBP, diastolic blood pressure; MBP, mean blood pressure; HR, heart rate; RMSSD, root-mean-square of successive differences; SDNN, standard deviation of normal-to-normal interval; LF/HF ratio, low-frequency/high-frequency ratio.

^a^Measured 5 min after the induction of anaesthesia.

ΔSBP was significantly greater in the GA than in the SA group (73.0 [26.0–86.1] *vs.* 2.0 [− 4.0 to 6.0] mmHg; median difference [95% CI], − 71.0 [− 84.0, − 30.5] mmHg; P < 0.001). ΔDBP, ΔMBP, and ΔHR were also greater in the GA group (ΔDBP: 42.5 [24.0–56.0] vs. 3.0 [− 3.0 to 6.0] mmHg; P < 0.001, ΔMBP: 42.9 ± 28.3 vs. − 1.4 ± 29.0 mmHg; P < 0.001, ΔHR: 18.6 ± 19.6 vs 3.5 ± 14.9 beats per min; P = 0.014). No patient showed an SBP elevation of more than 20% from baseline in the SA group.

The HRV analysis did not yield significant differences in HRV-related measures between the two groups (Table 2); however, RMSSD after bladder hydrodistention was significantly lower in the GA group (10.8 [7.7–19.8] *vs.* 20.6 [15.1–44.7] ms; P = 0.045).

Anaesthetic and operational findings are listed in Table 3. No patients in the SA group were administered rescue drugs, whereas 77.8% of patient in the GA group received rescue vasopressors during surgery (P < 0.001). There were no differences in surgery, anaesthesia, or PACU stay time.

**Table 3:** Comparison of anaesthetic and operational findings between the two groups.

	GA group (n = 18)	SA group (n = 18)	Median, mean, or proportion difference [95% CI]	P value
Bupivacaine dose (mg)	N/A	12.9 ± 1.4	N/A	N/A
Sensory block level^a^	N/A	T7 [T6–T8]	N/A	N/A
Volatile agent (MAC)^a^	0.9 [0.7–1.0]	N/A	N/A	N/A
Remifentanil concentration (ng/mL)^a^	5.3 [4.0–6.0]	N/A	N/A	N/A
Use of rescue vasopressor^b^	14 (77.8%)	0 (0.0%)	− 77.8% [− 91.0%, − 48.9%]	< 0.001
During induction	5 (27.8%)	0 (0.0%)	− 27.8% [− 50.9%, 4.5%]	0.018
Before bladder hydrodistention	6 (33.3%)	0 (0.0%)	− 33.3% [− 56.2%, − 8.8%]	0.008
After bladder hydrodistention	8 (44.4%)	0 (0.0%)	− 44.4% [− 66.2%, 17.9%]	0.002
Use of rescue vasodilator	5 (27.8%)	1 (5.6%)	− 22.2% [− 45.8%, 3.1%]	0.178
MAC during hydrodistention				
Remifentanil concentration				
Surgery time (min)	31.9 ± 12.0	32.2 ± 9.8	0.3 [− 7.1, 7.7]	0.928
Anaesthesia time (min)	57.2 ± 13.0	59.7 ± 11.4	2.4[− 5.8, 10.7]	0.551
Postoperative outcomes				
PACU stay time (min)	40.0 [39.0–41.0]	30.5 [24.0–56.0]	− 9.5 [− 15.5, 15.0]	0.193
Pain score in PACU (NRS)	3.5 [0.0–5.5]	0.0 [0.0–0.0]	− 3.5 [− 4.8, 0.0]	< 0.001
Use of analgesics in PACU	2 (11.1%)	0 (0.0%)	− 11.1% [− 32.8%, 8.2%]	0.486
Cystoscopic findings				
Hunner’s lesions	17 (94.4%)	15 (83.3%)	11.1% [− 11.8%, 34.1%]	0.596
Glomerulation	6 (33.3%)	3 (16.7%)	16.6% [− 11.7%, 42.0%]	0.441
Mucosal rainy bleeding	3 (16.7%)	10 (55.6%)	38.9% [7.5%, 61.5%]	0.037

GA, general anaesthesia; SA, spinal anaesthesia; N/A, not applicable; PACU, post-anaesthesia care unit; NRS, numeric rating scale.

^a^Measured before hydrodistention.

^b^In some cases, multiple doses of vasopressors were administered.

Pain scores in the PACU were significantly higher in the GA group (3.5 [0.0–5.5] *vs.* 0.0 [0.0–0.0]; P < 0.001). No patient required rescue analgesics in the SA group, whereas two patients were administered analgesics in the GA group (2 [11.1%] *vs.* 0 [0.0%]; P = 0.486).

In terms of cystoscopic findings, mucosal rainy bleeding was less frequently observed in the SA group (3 [16.7%] *vs.* 10 [55.6%]; P = 0.037).

The results of the subgroup analysis with respect to hemodynamic and autonomic responses are presented in Table 4. Six patients treated with rescue vasopressors before bladder hydrodistention, such as during anaesthesia induction, were excluded from the GA group. ΔSBP, the proportion of patients with an SBP increase of more than 20%, ΔDBP, ΔMBP, and ΔHR were all significantly greater and the RMSSD after bladder hydrodistention was still significantly lower in the GA than in the SA group.

**Table 4:** Subgroup analysis of hemodynamic outcomes between the two groups.

	GA group (n = 12)	SA group (n = 18)	Median, mean, or proportion difference [95% CI]	P value
Differences between max and baseline				
ΔSBP (mmHg)	77.5 [21.0–86.5]	2.0 [− 4.0 to 6.0]	− 75.5 [− 85.5, − 19.0]	< 0.001
SBP increase > 20%	9 (75.0%)	0 (0.0%)	− 75.0% [− 91.1%, − 41.7%]	< 0.001
ΔDBP (mmHg)	43.0 [17.5–55.5]	3.0 [− 2.0 to 6.0]	− 40.0 [− 53.0, − 13.5]	< 0.001
ΔMBP (mmHg)	56.0 [12.0–65.0]	1.0 [− 1.0 to 10.0]	− 55.0 [− 65.0, − 9.0]	0.001
ΔHR (beat per min)	22.6 ± 19.9	3.5 ± 14.9	− 19.1 [− 33.2, − 5.0]	0.006
HRV before bladder hydrodistention				
RMSSD (ms)	11.4 [6.1–15.2]	19.3 [13.2–29.4]	7.9 [− 0.9, 20.5]	0.080
SDNN (ms)	10.2 [6.5–23.8]	20.5 [15.2–32.8]	10.3 [− 3.9, 23.3]	0.285
LF/HF ratio	2.5 [1.3–6.8]	2.3 [0.5–12.8]	− 0.2 [− 5.1, 7.7]	0.849
HRV after bladder hydrodistention				
RMSSD (ms)	9.7 [7.1–12.5]	20.6 [15.1–44.7]	10.9 [4.2, 34.9]	0.010
SDNN (ms)	13.7 [8.4–18.0]	18.5 [11.8–38.3]	4.9 [− 4.3, 26.9]	0.238
LF/HF ratio	2.4 [0.4–14.1]	1.4 [0.4–3.5]	− 1.1 [− 13.2, 1.5]	0.495

GA, general anaesthesia; SA, spinal anaesthesia; SBP, systolic blood pressure; DBP, diastolic blood pressure; MBP, mean blood pressure; HR, heart rate; RMSSD, root-mean-square of successive differences; SDNN, standard deviation of normal-to-normal interval; LF/HF ratio, low-frequency/high-frequency ratio.

## Discussion

In this study, we demonstrated that SA has some advantages over GA in preventing abrupt hemodynamic responses during bladder hydrodistention in patients with IC/BPS. In particular, an SBP increase of more than 20% from baseline was only observed in the GA group. Moreover, the number of patients requiring rescue vasopressors was smaller and postoperative pain scores in PACU were lower in the SA group.

In healthy individuals, as the hydrostatic pressure rises during bladder filling, afferent Aδ fibres in the hypogastric and pelvic nerves increase their activity to stimulate the sympathetic nervous system, resulting in relaxation of the bladder smooth muscles^19^. However, in previous studies using the IC/BPS animal model, C-fibre sensitized by chronic inflammation of the bladder showed hyperexcitability with a reduced threshold, resulting in increased sympathetic tone^20^. This mechanism explains the hike in blood pressure accompanied by increased urine norepinephrine and decreased vagal activity in patients with IC/BPS^21, 22^. The results of the present study demonstrated that spinal anaesthesia, which produces intense blockade of neuronal transmission, could have advantages compared to general anaesthesia for blocking autonomic responses. These findings are also consistent with the results of the previous retrospective study^10^.

HRV is a widely used physiologic test to evaluate the imbalances within the autonomic nervous system. According to the results of the present study, measures of HRV did not differ between the groups before bladder hydrodistention. However, RMSSD after bladder hydrodistention was significantly lower in the GA group. RMSSD reflects the vagal-mediated changes in HR^23^. These results may thus be due to the increased sympathetic tone after bladder hydrodistention in the GA group. A previous study has also reported lower RMSSD in patients with chronic pain^24^.

Postoperative pain increases recovery times and the length of hospital stays, and lowers patient satisfaction^25, 26^. Previous studies conducted in patients undergoing other urologic procedures have also reported that spinal anaesthesia is superior to general anaesthesia in terms of higher patient satisfaction, shorter recovery times, and lower levels of postoperative pain^27, 28^. In the present study, patients in the SA group neither experienced postoperative pain nor required rescue analgesics in the PACU. However, our follow-up period was short, not reflecting the full extent of postoperative pain. Therefore, although SA was superior for short-term postoperative pain after hydrodistention in this study, further research based on long-term follow-up is needed.

Although spinal anaesthesia has several advantages over general anaesthesia during bladder hydrodistention, there are possible caveats to spinal anaesthesia; particularly, bladder rupture may occur due to adductor muscle spasms triggered by the stimulation of the obturator nerve during electrocautery for coagulation. A previous study observed an obturator reflex in 20% of patients under GA; such a case was, however, not observed in this study^29^. Obturator nerve block might be considered in patients at high risk of bladder injury due to adductor muscle spasm. In addition, in this study, mucosal waterfall bleeding was more frequent in the SA group, which may have been the result of spinal anaesthesia-induced vasodilation in the lower body.

This study has several limitations. First, we determined the remifentanil concentration (4–6 ng/mL) prior to bladder hydrodistention in the GA group based on the remifentanil concentration required for preventing hemodynamic responses to tracheal intubation in a previous study^30^. However, this remifentanil concentration was not sufficient to block autonomic responses during bladder hydrodistention under GA in our IC/BPS patients. Nevertheless, a further increase in remifentanil dose can cause more decrease in systolic blood pressure before hydrodistension in the GA group. Future studies on effective strategies (e.g.*,* short acting vasodilator administration) for preventing abrupt increases in SBP during bladder hydrodistention under GA are needed. Second, we did not evaluate postoperative pain in the general ward. There is, however, a possibility of rebound pain arising after spinal anaesthesia. Third, we did not compare long-term outcomes, such as recurrence and symptom improvement, for the two anaesthetic techniques.

In conclusion, we found that spinal anaesthesia has some advantages over general anaesthesia for bladder hydrodistention in terms of preventing an abrupt increase in SBP and controlling pain in the PACU.

## Supplementary Information

Supplementary Information.

## Data Availability

The datasets generated during and/or analysed during the current study are available from the corresponding author on reasonable request.
